# Sterile Injury Repair and Adhesion Formation at Serosal Surfaces

**DOI:** 10.3389/fimmu.2021.684967

**Published:** 2021-05-14

**Authors:** Simone N. Zwicky, Deborah Stroka, Joel Zindel

**Affiliations:** Department of Visceral Surgery and Medicine, Department for BioMedical Research (DBMR), University of Bern, Bern, Switzerland

**Keywords:** peritoneal adhesions, peritoneum, sterile injury, mesothelium, post-surgical adhesions

## Abstract

Most multicellular organisms have a major body cavity containing vital organs. This cavity is lined by a mucosa-like serosal surface and filled with serous fluid which suspends many immune cells. Injuries affecting the major body cavity are potentially life-threatening. Here we summarize evidence that unique damage detection and repair mechanisms have evolved to ensure immediate and swift repair of injuries at serosal surfaces. Furthermore, thousands of patients undergo surgery within the abdominal and thoracic cavities each day. While these surgeries are potentially lifesaving, some patients will suffer complications due to inappropriate scar formation when wound healing at serosal surfaces defects. These scars called adhesions cause profound challenges for health care systems and patients. Therefore, reviewing the mechanisms of wound repair at serosal surfaces is of clinical importance. Serosal surfaces will be introduced with a short embryological and microanatomical perspective followed by a discussion of the mechanisms of damage recognition and initiation of sterile inflammation at serosal surfaces. Distinct immune cells populations are free floating within the coelomic (peritoneal) cavity and contribute towards damage recognition and initiation of wound repair. We will highlight the emerging role of resident cavity GATA6+ macrophages in repairing serosal injuries and compare serosal (mesothelial) injuries with injuries to the blood vessel walls. This allows to draw some parallels such as the critical role of the mesothelium in regulating fibrin deposition and how peritoneal macrophages can aggregate in a platelet-like fashion in response to sterile injury. Then, we discuss how serosal wound healing can go wrong, causing adhesions. The current pathogenetic understanding of and potential future therapeutic avenues against adhesions are discussed.

## Development and Microanatomy of the Coelom and Mesothelium

**Figure 1:**
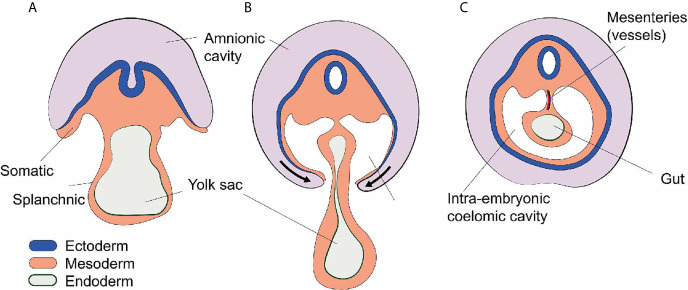
Development of the intra-embryonic coelomic cavity. **(A)** Schematic cross section human embryo of 3 weeks age. The mesoderm shows a somatic (dorsal) and splanchnic (ventral) aspect. **(B)** Cranio-caudal and latero-lateral folding in week 4. **(C)** After closure of the anterior abdominal wall the intra-embryonic coelomic cavity is formed. Organs (e.g. gut) are suspended by dorsal and sometimes ventral (not shown) mesenteries carrying blood vessels and nerves.

During embryology, at the end of the third week, the lateral plate mesoderm is divided into two layers: the somatic and splanchnic mesoderm layer (1). These two layers form a cleft that becomes a cavity as the embryo undergoes a cranio-caudal and latero-lateral folding event in week four (1). This cavity is called the intraembryonic coelom and contains vital organs such as the heart, the lungs, the liver, and the intestines. In mammals, the mesodermal lining of the coelom differentiates into a serous epithelium-like membrane called mesothelium (2). The somatic mesoderm gives rise to the parietal layer of the mesothelium which lines the body wall, and the splanchnic mesoderm gives rise to the visceral layer of the mesothelium which lines the surfaces of organs. The intra-coelomic organs stay connected to the body wall by elongations referred to as mesenteries which contain blood vessels, lymphatics, and nerves (1) (**Figure 1**).

Later, the coelomic cavity is further subdivided resulting in three embryologically related but anatomically distinct anatomical compartments: the pericardial cavity, the pleural cavities, and the peritoneal (abdominal) cavity. All of these contain vital organs such as heart, lung, and abdominal organs (1).

**Figure 2:**
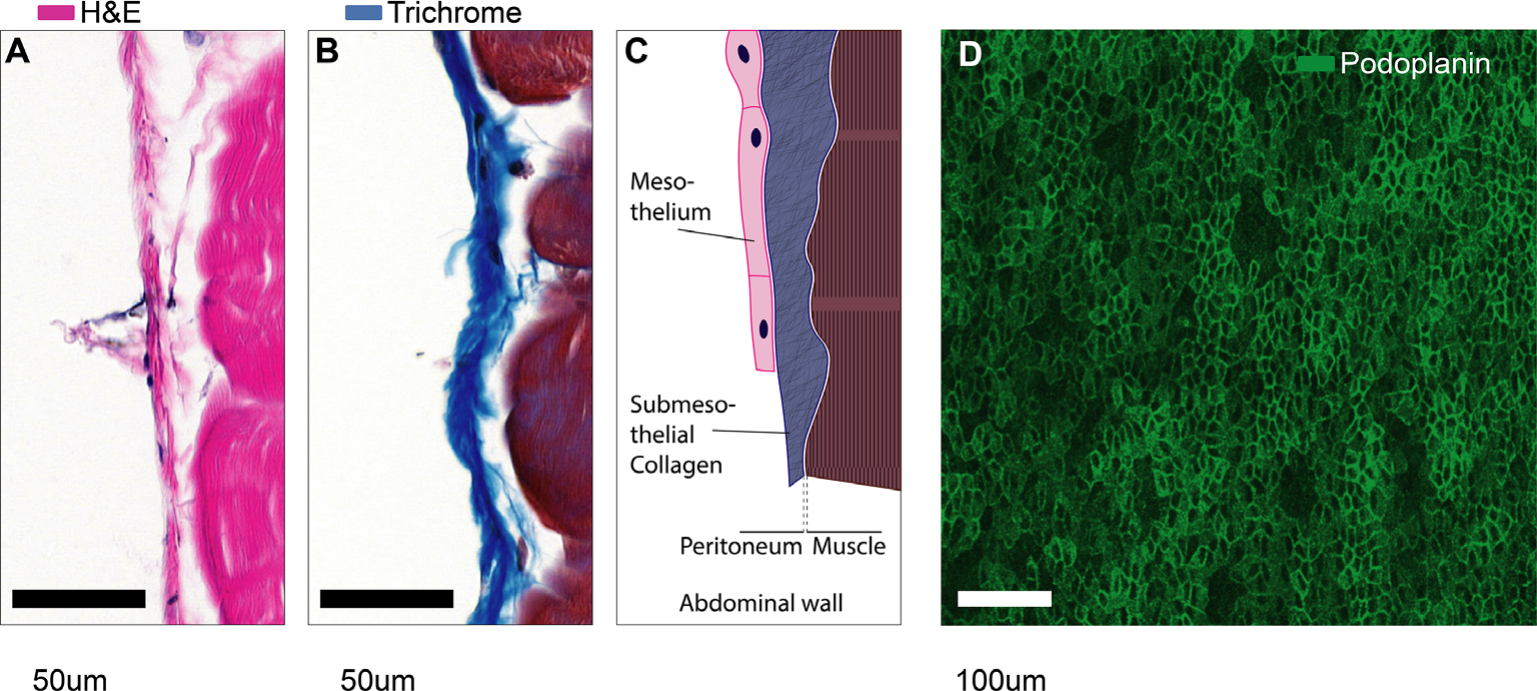
Microanatomy of mesothelial surfaces. **(A, B)** Cross sections of mouse abdominal wall stained with Hematoxylin & Eosin **(A)** and Masson’s trichrome staining **(B)**. Scale bars: 50 µm. **(C)** Illustration of the structures shown in **(A, B)**. **(D)** Top view on mesothelial surface stained with anti-podoplanin antibody.

The serous membrane that covers the walls of all coelomic cavities as well as the borders of all organs contained within them is also called the serosa and is comprised of a flat monolayer of mesothelial cells. The serosal linings ensure friction-less movement of organs and establish a water-tight barrier separating the fluid-filled cavities from surrounding tissues (**Figure 2**). Together with the associated sub-mesothelial connective tissue the serosa is also called peritoneum, pleura, and pericardium in the peritoneal (abdominal), pleural and pericardial cavities, respectively. In practice, the terms mesothelium, serosa, and peritoneum (or pleura or pericardium) are often used interchangeably.

The peritoneum is less than 25 µm thick in the mouse (3) and about 50-100 µm thick in humans (4, 5). Therefore, as we discuss injury at serosal surfaces, it is important to note that the mesothelium will rarely be injured in an isolated fashion. In fact, serosal injuries will often compromise the tissues that are covered by the mesothelium as well. These underlying tissues can be vastly different such as:

- smooth-muscular wall of the intestines, urinary bladder, uterus,
-parenchymal tissue of heart, lung, liver, gallbladder, spleen (only mouse), ovaries,
- fat tissue of the omentum,
- striated muscle, fascia, and bone of the thoracoabdominal wall and diaphragm,
- and connective tissues such as that of the pericardium.

Any experimental model system that studies serosal wound repair, may invoke some underlying tissue-specific wound repair mechanisms. This review is targeted at serosa specific mechanisms, but we ask the reader to bear in mind that we use the generalization “at serosal surfaces” inductively; some of the mechanisms discussed here may apply only to specific locations within the coelomic cavity.

## Cells Suspended in Coelomic Cavities

**Figure 3:**
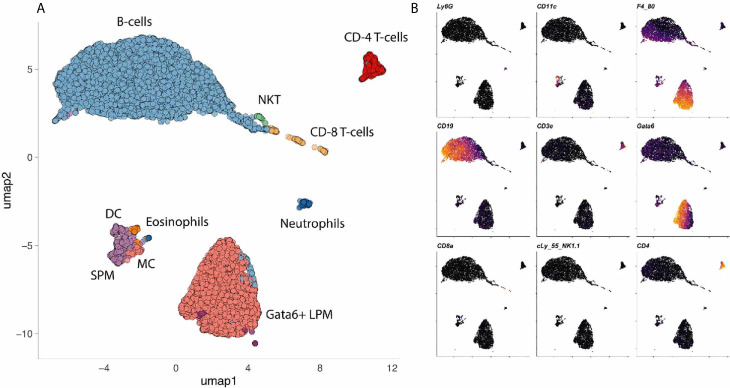
Cells in a mouse coelomic cavity. **(A, B)** Peritoneal cavity lavage of healthy C57Bl/6 mice. Dimensionality reduction dimension 1 (umap1) and 2 (umap2) of myeloid lineage markers (mass cytometry) are plotted on x- and y-axis, respectively. Dots (cells) are colored by cluster **(A)** or marker **(B)**. Data with kindly permission from M. Dosch and G. Beldi.

The coelomic cavities are filled with fluid that suspend millions of cells also referred to as coelomocytes. The coelomocyte composition of mice and humans has been reviewed elsewhere (6). Briefly, the human peritoneal cavity suspends a total of 10^7^ leukocytes in 5-100ml of peritoneal fluid (6, 7). In mice, the number of peritoneal leukocytes varies between strains from 3 to 5x10^6^ cells (8). The pleuropericardial cavities contain 0.3-1x10^6^ leukocytes per mouse (6, 9, 10). Most leukocytes in the peritoneal cavity are lymphocytes (10-60%) and macrophages (40-60%) (8, 11–16). In addition, the peritoneal cavity contains dendritic cells (2 – 6%) (12, 17), mast cells, eosinophils, neutrophils (0-31%), innate lymphoid cells (ILCs) including natural killer cells and mesothelial cells (14, 16) (**Figure 3**).

In terms of wound healing, the role of peritoneal macrophages is best established. Macrophages make up 40-60% of all coelomocytes in both mice and humans. Two major subpopulations of peritoneal macrophages have been described (14). The small peritoneal macrophages (SPM) are monocyte-derived, constantly replenished and can be recruited within hours in significant amounts (14). At baseline, they account for about 5% of all immune cells or about 10% of all macrophages (14, 18). The majority (90%) of peritoneal macrophages belong to a distinct tissue-residential macrophage population. Since these resident cells are slightly larger than their monocyte-derived sisters, they are also referred to as large peritoneal macrophages (14). The large peritoneal macrophages (LPM) are a self-renewing population characterized by the expression of CD102 (Icam2), high levels of F4/80 and the transcription factor GATA6 (19–22). GATA6+ LPM seem to be well conserved when comparing the different coelomic cavities of mice and human (23–25). Canonically, these GATA6+ cavity macrophages are thought to clear bacteria by phagocytosis (14, 26) and also by inducing intra-abdominal formation of fibrin clots that immobilize bacteria (21). In primordial species such as the purple sea urchin (*Strongylocentrotus purpuratus*), coelomocytes are also crucial for tissue repair, in addition to clearing toxins and pathogens (27–30). The importance of GATA6+ cavity macrophages in damage recognition and tissue repair will be discussed in detail.

## Damage Recognition and Inflammation

Wound repair at large starts with inflammation. Inflammation is induced when a significant deviation from homeostasis is detected. According to the current paradigm, such a deviation could be the presence of microbes (infection) or damaged tissue (injury). The innate immune system has developed an effective arsenal of surveillance cells that constantly probe their microenvironment for deviations from homeostasis. On a molecular level, deviation from homeostasis is defined by the occurrence of pre-specified molecular patterns. Immunostimulatory molecular patterns that induce inflammation in case of sterile injury, i.e., in the absence of pathogens and their products, have been termed damage associated molecular patterns (DAMPs). DAMPs have been extensively reviewed elsewhere (31). In brief, DAMPs comprise different molecules that are not normally present outside of cells such as double stranded DNA, nuclear proteins, mitochondrial DNA, mitochondrial proteins, and molecules with high cytosolic concentrations such as ATP or K^+^ Ions. In addition, damaged cells may induce the production and release of additional DAMPs (iDAMPs) such as heat shock proteins, defensins, galactins and interleukin 1 (IL-1). Furthermore, if proteins that are constitutively present in the extracellular space such as hyaluronan, biglycan, heperansulfate and other extracellular matrix (ECM) components are modified by injuries, they can also become DAMPs. Under homeostatic conditions, the serosal surfaces are covered with glycoconjugates such as sialomucins, hyaluronic acid, and glycoproteins like fibronectin (32–35). These molecules contain large anionic sites that cover the serosal surfaces with a negatively charged coat—also referred to as the glycocalyx—that may help to repulse invading microbes (32) and ensure friction-less movement of intra-coelomic organs (35). The loss of this negatively charged coating due to serosal injury, may serve as mesothelium-specific DAMP or “touch me signal” (36).

Molecules that allow eukaryotic cells to detect the presence of DAMPs have been termed pattern recognition receptors (PRR). The expression of PRR such as toll-like receptors 1 through 6 (TLR-1-6), nucleotide-binding oligomerization domain (Nod)-1 and Nod-2 and advanced glycation end product (AGE) receptors, has been demonstrated for murine and human mesothelial cells (37). Upon activation, mesothelial cells release cytokines and inflammatory mediators such as chemokine (C–C motif) ligand 2 (CCL2), CCL5, (C–X–C motif) ligand 8 (CXCL8), and nitric oxide (38, 39). Furthermore, mesothelial cells upregulate adhesion molecules that presumably facilitate the migration of inflammatory leukocytes across and along serosal surfaces. These include intercellular adhesion molecule-1 (ICAM-1), vascular cellular adhesion molecule-1 (VCAM-1), E-cadherin, N-cadherin, CD29 and CD44 (38, 40–42). It is important to note that cellular adhesion molecules expressed by mesothelial cells play a dual role in serosal wound repair. While an initial upregulation may facilitate leukocyte recruitment, these molecules, especially E-cadherin, are downregulated later during serosal wound repair. The latter is associated with loss of mesothelial cohesion enabling the mesothelium to switch to a more mesenchymal program, a process that we will discuss in detail below. In addition, mesothelial cells modulate inflammation by synthesis and release of hyaluronan (43), which is able to sequester free radicals and initiate tissue repair responses (38).

*In vivo*, the initiation of inflammation at serosal surfaces does not rely on mesothelial cells alone but on a series of events. These comprise specialized cellular and humoral immune mechanisms such as leukocyte recruitment, complement activation and production of natural antibodies. In the rest of this chapter, we will discuss these elements one by one.

### Mesothelial Damage Is First Recognized by Cavity Macrophages

Recent advances in intravital microscopy have allowed to characterize the sequence of cells recruited to mesothelial injuries. By using resonant-scanners, multi-photon excitation, and extremely sensitive hybrid detection systems it became possible to image the peritoneal cavity through the intact abdominal wall under real-life conditions (21, 44). Second, multi-photon imaging allows the use of near-infrared microscopy lasers to induce focal thermal injuries during intravital microscopy with high precision (44–46). By combining intravital microscopy of the abdominal cavity with peritoneal laser injuries, we were able to image cellular recruitment to mesothelial injuries. Surprisingly, the first GATA6+ cavity macrophages attached at the injuries within only a few seconds and the macrophages completely covered the lesions after 15 minutes of imaging (44). The recruitment of cavity macrophages to mesothelial injury was significantly faster than that of neutrophils, which needed much longer (> 40 minutes) (44). Cavity macrophages were present in the peritoneal fluid in vast numbers and traversed the peritoneal cavity in a seemingly random fashion within respiration-dependent movement of peritoneal cavity content (44). The observations that these cells seemed to rely on passive transportation by peritoneal fluid, and that they—upon contact with cell already adhering to the injury—were forming stable cell-cell aggregates were very reminiscent of the platelet aggregation that took place when a nearby blood vessel wall was damaged using laser injury. We concluded that cavity macrophages randomly “patrol” the serosal surfaces in a platelet-like fashion and rapidly form aggregates in response to DAMPs. This is consistent with a previous electron microscopy study by Haney showing that peritoneal macrophages invariably detected and migrated to injuries of the peritoneal membrane (47). In addition, Wang and Kubes showed that cavity macrophages were able to detect mesothelial injuries of the liver capsule and migrated to the injured liver (36). On a molecular level, this interaction occurred independent of integrins or selectins, instead peritoneal macrophages relied on different receptor molecules such as macrophage receptor with collagenous structure (MARCO), Macrophage scavenger receptor 1 (MSR1), CD44, and purinergic receptor P2X7. The respective DAMPs recognized by CD44 and P2X7 are hyaluronan and ATP respectively (36). The ligands that mediate MARCO and Msr1 dependent macrophage aggregation are yet to be identified (44).

The function of peritoneal macrophages in sterile injury is multi-facetted. Current models indicate that ligation of DAMPs to PRR on macrophages leads to their inflammatory polarization—also referred to as M1 polarization. This activation would result in the production of pro-inflammatory cytokines such as tumor necrosis factor (TNF) and IL-1 (31, 48). However, peritoneal macrophages recruited to sterile liver injury were shown to skew their phenotype towards alternative or repair polarization—also referred to as M2 macrophages—increasing their expression of CD273, CD206 and Arginase 1 (36). Interestingly, Uderhardt et al. recently investigated the resident tissue macrophages of the muscular abdominal wall. The abdominal wall macrophages are distinct from the peritoneal cavity macrophages suspended in the peritoneal cavity. They proposed that abdominal wall macrophages can extend their pseudopods toward local injury sites within a radius of 100-150µm. In their study, resident tissue macrophages were able to completely enclose lesions if their size was below a certain threshold (microlesions). This—as the authors termed it—cloaking mechanism, was able to block scouting neutrophils from interacting with DAMPs and thus prevented subsequent neutrophil driven inflammation and tissue destruction (45). The cloaking mechanism was described for tissue resident macrophages in the muscular abdominal wall, i.e., on the far side of the mesothelium with respect to the coelomic cavity. It needs to be determined whether scavenger receptor mediated macrophage aggregation on the coelomic site of the mesothelium causes inflammation or whether aggregation of cavity macrophages serves to contain injuries and is therefore—in essence—anti-inflammatory. Ultimately, aggregation of peritoneal cavity macrophages in response to mesothelial injuries was shown to improve tissue repair (36, 44, 47).

### Cavity Macrophage Disappearance Reaction

Aggregation of peritoneal macrophages causes their number in the peritoneal lavage to drop. The decrease in their number was correlated with the injury size (44). With larger injuries of the mesothelium, such as a surgical laparotomy, the number of GATA6+ cavity macrophages in the peritoneal lavage was reduced to zero (44). In other words, these cells disappeared from the peritoneal fluid (lavage). However, this was not the first time, the sudden absence of macrophages was observed. In fact, over half a century ago, Nelson and Boyden described a sharp decline of macrophage count in peritoneal exudates in response to a hypersensitivity reaction to tuberculin in Bacille Calmette-Guérin (BCG)-vaccinated guinea pigs. They termed this the “macrophage disappearance reaction” (MDR) (49). Since then, various insults (sterile and microbial) to the peritoneal compartment have been found to induce the MDR (**Table 1**).

**Table 1:** Macrophage disappearance reaction (MDR). Studies describing MDR from 1963 until now.

MDR Trigger (dose)	Time between trigger and complete MDR	Postulated fate of disappeared macrophages	Molecular mechanism	Reference
**Sterile Models**				
Sterile mesothelial injury (surgery, laser)	3h	Form stable cell-cell aggregates that cover injury and induce post-surgical adhesions	Scavenger receptors, can be blocked with Heparin and Poly-(I)	(44)
Sterile Brewer’s Thioglycollate	12-72h	Macrophage cell death	Not demonstrated	(50–52)
Antigen, migration inhibitory factor, viruses or tumor cells	1 to 96h	Undergo activation during MDR in delayed type hypersensitivity or acute inflammatory reaction and then reappear activated to regulate responses toward pathogens or tumor cells.	MDR Inhibited by Heparin, L-Fucose, Hyaluronidase	(53)
Egg Antigen (10ug), purified protein derivate (10ug)	5h	Macrophage activation	Desensitization suppress MDR, in sensitized animals normal MDR	(54)
Tuberculin	2.5 - 6h	Not demonstrated	MDR completely inhibited by Heparin and Warfarin	(49, 55)
Ova peptide (50ug) into mice bearing antigen-primed T cells	5h	Macrophage adhesion	Suppressed in fibrinogen- deficient mice, partially suppressed by thrombin antagonist	(56)
Thrombin (20 Units)	1h -5h	Macrophage adhesion	MDR suppressed in fibrinogen-deficient mice.	(56)
RGES Peptide	48h	Macrophage bind the mesothelium overlying draining lymphatics	Integrin-mediated mechanisms involving VLA-4 and VLA-5 that can be blocked by RGD (Arg-Gly-Asp peptides) and VLA-4 and VLA-5 blocking antibodies.	(57)
**Microbes or microbial products**				
E. coli (5×10^7^ UV-inactivated)	20d	Do not undergo fas-mediated apoptosis	No difference in fas-deficient mice.	(58)
S. aureus	2h (2 × 10^7^)	Not demonstrated	Not demonstrated	(59)
Lipopolysaccharide	3h (10 µg) 5h (1 µg)	Accumulation in the omentum	Macrophage interaction with mesothelial cells, mainly of the omentum, was proposed to be a key step in MDR. Partially inhibited by refludan.	(19, 40, 56)
Zymosan	3-4h (1mg) 4h (0.5mg) 3d (10 µg)	Form large clots to trap microorganisms; adherence with tissue and drained to lymph node	MDR reversed completely with Heparin and partially with Hirudin/loss Factor V Expression/loss of Integrin activation adaptor talin-1 Expression/TF deficiency	(21, 51, 60–62)
INF-γ (100 U/mL) + LPS (100ng/ml)	20h	Binding to mesothelial cells	Monocyte activated by *in vitro* exposure to LPS and INF-Y bound with increased efficiency to mesothelial cells	(40)
Synthethic Lipopetid (Pam3CSK4)	12h	Not demonstrated	Not demonstrated	(59)
**Human studies**				
Bacterial peritonitis	1 day	Shedding of surface CD206	Depletion of CD206+ LPM at day 1 of SPB Peritonitis with normalization to steady state after resolution of SPB	(63)
Liver cirrhosis associated events (Bacterial peritonitis, encephalopathy, death)		Not demonstrated	Severity of liver disease and liver cirrhosis are correlated with reduced numbers of CrIg^hi^ macrophages. Human CrIg^hi^ macrophages were transcriptionally similar to mouse F4/80hi peritoneal macrophages.	(25)

These studies indicate that the MDR is not a specific reaction but arguably follows any inflammatory challenge to the peritoneal compartment. While some reports indicate that peritoneal macrophages can leave the peritoneal cavity through the draining lymphatics (52, 60, 64), most of the more recent reports suggest that peritoneal macrophages have the tendency to adhere to each other (aggregate) as well as to the mesothelium in response to challenge (**Table 1**). Therefore, the loss of dispersion and cellular aggregation are a commonality among the different models of MDR. The MDR correlates with increased inflammatory cytokine levels in the peritoneal fluid and the influx of pro-inflammatory leukocytes such as monocytes, eosinophils, and neutrophils into the peritoneal compartment (21, 59). Cailhier et al. used CD11b driven diphtheria toxin receptor and low dose intraperitoneal injections of diphtheria toxin to selectively deplete resident peritoneal macrophages. In an experimental peritonitis model, this resulted in a significant decrease of inflammation (infiltration of neutrophils) that could be restored by the adoptive transfer of resident, non-transgenic, peritoneal macrophages (65). These data indicate that the aggregation of cavity macrophages in response to a strong stimulus, such as peritonitis, causes inflammation. However, in the case of smaller insults such as focal injuries or localized microbial challenges, MDR may compartmentalize the insult, in analogy to the cloaking mechanism described for macrophages of the muscular abdominal wall (45). Along those lines, complete MDR could be interpreted as a threshold above which all macrophages have been “used up” indicating that the attempt at cloaking the insult has failed, which in turn results in inflammation. Either way, it would be important to study the largely unknown (intracellular) changes in macrophages undergoing a disappearance reaction in sterile and microbial models.

### Dendritic Cells and Mast Cells

The peritoneal cavity harbors CD11c^+^ dendritic cells as well as cKit^+^ mast cells both of which are canonical initiators of inflammation. Their role as antigen presenting cells and inducers of inflammation in response to bacterial infection is well documented. In fact, CD11c+ dendritic cells are required for survival in murine polymicrobial peritoneal sepsis (66). In addition to pathogen-derived ligands for PRR, several DAMPs have been shown to interact with dendritic cells and dramatically affect their function (67, 68). Interestingly, the response of dendritic cells to DAMPs is not always clear-cut, with different responses depending on dendritic cell subtypes and location (67). For example, activation of dendritic cells in sterile liver injury leads to the secretion of anti-inflammatory cytokines such as IL-10 and TGF-β (67) while similar injury models of kidney and gut may lead to a pro-inflammatory response and secretion of IL-6, IL-12 and TNF-α (67, 69). So far, the response of peritoneal dendritic cells to serosal injury is not well understood and requires further studies. Mast cells have traditionally been studied in the context off helminthic infections and Ig-E mediated reactions. It becomes clear, that mast cell degranulation is also an important modulator of wound healing of skin wounds (70) and lesions in the gastrointestinal tract (71–73). Poerwosusanta et al. investigated the role of mast cell degranulation in mesothelial injury. Mesothelial injury was carried out by performing laparoscopic surgeries in rats at different intra-abdominal inflation pressures (74). They showed that an increased intraabdominal pressure—and presumably increased stress to the mesothelium—led to an increased number of mast cells that infiltrated the mesothelium. This was correlated with increased mast cell degranulation. This increased mast cell count is consistent with findings from skin injury models and is due to chemokine-dependent mast cell immigration rather than local proliferation. More detailed investigation, e.g. based on intravital microscopy, could help to elucidate whether mast cells are recruited to mesothelial injuries by blood or directly from the peritoneal cavity.

### Humoral Pattern Recognition Molecules and Natural Antibodies

The fluid of the pleural and peritoneal cavity in mice and humans not only contains cells but also large amounts of proteins of the coagulation system and complement system as well as large amounts of natural antibodies (75, 76). In the peritoneal and pleural cavities, the complement proteins are produced by mesothelial cells (75, 77). The complement system is an ancient enzymatic cascade of proteins with the main function of opsonization and lysis of bacteria (78). The alternative pathway of the complement can be activated by injuries and in the last decade a role of complement activation in wound healing (79) and regeneration (80, 81) as well as morphogenetic and developmental processes (82, 83) has been suggested. Furthermore, some clinical studies have evaluated the role of blood complement during major surgery and described a correlation of the invasiveness of the procedure with the amount of complement used, indicating that sterile injury leads to complement activation in humans (84). Inversely, humoral molecules that are canonically associated with innate immunity, have been shown to mediate tissue repair and regulate fibrosis. For example, pentraxin 3 (PTX3) was shown to reduce fibrin deposition and fibrosis in several wound models outside of the peritoneal cavity. While to our knowledge, no humoral molecule with anti-fibrotic properties has been described in the peritoneal cavity, the discovery of such could have great therapeutic potential (85–87).

Like complement factors, natural antibodies are abundant in the coelomic cavity fluids and are primarily thought to combat microbes by recognizing a wide variety of different microbial antigen patterns (88). The peritoneal natural antibodies are mainly produced by self-replenishing peritoneal B1 cells in an antigen-independent manner (89). The repertoire of natural antibodies also enables recognition of self-antigens such as phosphorylcholine, phosphatidylcholine and carbohydrate determinants. Furthermore, natural antibodies have been shown to sense apoptotic cells (88) and electronegative DAMPs (90). In addition, natural antibodies were able to accelerate wound healing by recruiting additional wound macrophages (91). Along those lines, Grönwall et al. postulate that natural IgM antibodies are part of a synapse between an apoptotic cell (that binds IgM) and the phagocyte. This synapse is mediated by complement and complement receptors expressed by phagocytes (92). There is increased interest in studying the role of natural IgM, and IgM-dependent, complement-mediated phagocytosis in several disease models (93–95). Although available data is limited, it is conceivable that complement and natural antibodies of body cavities play an important a role in wound repair at serosal surfaces.

## Coagulation and Fibrin Deposition

It is widely accepted that inflammation of the peritoneal, pleural, or pericardial compartment, is associated with fibrin exudates. After mesothelial injury, the resulting inflammation as well as the injury itself, lead to the activation of tissue factor pathway and the inhibition of the antithrombin-III (AT-III) pathway (96). This is followed by the spontaneous cleavage of fibrinogen and cross-linking of fibrin. In addition to inflammation, injury causes the destruction of and consequent leakage from blood vessels of the abdominal wall. Extravasation of blood results in the activation of the canonical coagulation cascade which further enhances the deposition of fibrin (96). In vivo, the deposition of fibrin is limited at serosal surfaces by activated plasmin which continuously degrades fibrin. Plasmin levels are regulated by plasminogen activators tPA and uPA and their respective plasminogen activator inhibitors 1 and 2 (PAI 1, PAI 2) (96). Homeostatic mesothelial cells produce large amounts of Plasmin, uPA and tPA and low amounts of PAI1 and PAI2. Therefore, the deposition of fibrin at serosal surfaces is tightly controlled. During inflammation however, the mesothelial production of PAI1 and PAI2 is significantly increased resulting in a mesothelial program switch from fibrinolytic towards anti-fibrinolytic state, resulting in the visible deposition of fibrinous exudates (96).

The formation of a stable fibrin clot—also referred to as fibrin matrix or cross-linked fibrin—serves as the scaffold for the subsequent wound repair (granulation) tissue. This includes the infiltration of leukocytes and mesenchymal precursors and results in the deposition of ECM, ingrowth of vessels and nerves and finally, the re-mesothelialization of the injured serosa. If these fibrin clots grow too large, they can be the starting point for an abdominal adhesion, a pathology we will discuss in more detail below. See the Perspective **Box 1** speculating on parallels between vascular and mesothelial coagulation control.

Box 1 **Perspective.**

We find it intriguing that fibrin clots after surgery are not focally limited to sites of injury but seem to be formed at distant sites as well while other regions of the peritoneal cavity appear to be protected. During sepsis, spontaneous disseminated intravascular coagulation (DIC) is a major clinical problem. The distribution of fibrin clot deposits during DIC is poorly understood. Interestingly, the response of mesothelium and endothelium to inflammation share certain similarities and both lead to the spontaneous formation of disseminated fibrin clots. Studying the analogy between these two pathologies, that both involve an epithelial-like monolayer of mesodermal origin, may lead to the identification of common patterns and molecules governing the respective phenomena of DIC and peritoneal adhesions.

## Recruitment of Neutrophils and Monocytes

We discovered that the first cells recruited to mesothelial injuries are the peritoneal cavity macrophages (44). These cells reside suspended in the peritoneal fluid (19) and are recruited directly from their suspensive state to the mesothelium in case of injury. This comprises a special case of leukocyte recruitment that is unique to coelomic cavities. The canonical route of leukocyte recruitment is from the blood stream. The processes of leukocytes leaving the blood stream have been referred to as leukocyte adhesion cascade and trans-endothelial migration. The underlying mechanisms have been revisited and reviewed most comprehensively by Nourshargh et al. (97, 98). In perfused organs such as muscle or liver, neutrophils are recruited within 30 minutes to the inflammatory site from the bloodstream (46, 99). In the mesothelium, neutrophils are the first cells that arrive after peritoneal macrophages, after about 40-60 minutes (44). In response to focal sterile injuries, neutrophils show an extremely high degree of coordination. While intravascular chemokine gradient seems to be very important for successful directional migration over the initial distance within the vessel (31, 99), different molecules are the most potent chemotactic stimuli once the neutrophils are close to the wound (31, 99, 100). This has been demonstrated for molecules such as N-formyl peptides, ATP, and leukotriene B4 (LTB4) and their respective receptors on neutrophils called formyl peptide receptor, P2Y2 receptor and LTB4 receptor (31, 46, 99–102). These so-called gradients and autocrine feedback loops (LTB4) have been established for solid organs such as the liver or muscle. Whether neutrophils rely on similar mechanism to reach serosal surfaces has not been demonstrated yet but the technical advances of the last years will now allow us to address these questions using intravital microscopy.

Although the canonical role of neutrophils is to clear microbes, several reports suggest that they are imperative for timely restoration of tissue architecture after sterile injury by clearing necrotic material (99) and producing growth factors such as transforming growth factor β (TGF-β) and vascular endothelial growth factor (VEGF) (103–105). Furthermore, once neutrophils have cleared all necrotic tissue, they starve each other of DAMPs. This leads to neutrophils becoming apoptotic and cleared by macrophages, which is another critical step for tissue repair as it enables macrophages within the wound to switch from an inflammatory to an anti-inflammatory, pro-resolution program (99, 103, 106). In this environment, pro-resolution macrophages and neutrophils start to produce factors that inhibit the recruitment of additional neutrophils and enhance pro-resolution properties of macrophages. These factors include lipoxin A4, resolvins, protectins (107), phosphatidylserine containing microvesicles shed by neutrophils, chemokine sequestration through CCR5 modulation by neutrophils (108), Annexin A1 released from neutrophil granules or in microvesicles (109) and IL-10 (110). Taken together, neutrophils play an important role in clearing debris and setting up a pro-resolution environment.

The second major leukocyte population recruited to mesothelial injuries comprises inflammatory monocytes and monocyte-derived macrophages. It is generally accepted that the recruitment of neutrophils precedes the recruitment of monocytes in sterile injury (31, 111) but it remains controversial whether neutrophils recruitment is a necessary prerequisite for subsequent monocyte recruitment (112, 113). Rather than being pre-determined, the fate of recruited monocytes appears to be largely dependent on the environment (62). In the context of sterile injury repair, monocytes have been shown to differentiate into mature macrophages at the site of injury. This process has been reviewed before (114). In brief, Ly6C^hi^ monocytes are recruited to the wound, where they gradually differentiate into Ly6C^low^ macrophages. One of the molecules necessary for this conversion is nuclear receptor subfamily 4, group a, member 1 (Nr4a1) (115). In the peritoneal cavity, the influx of inflammatory monocytes due to injury or infection, correlates with the increase of MHCII^+^ CD102^-^ GATA6^-^ macrophages (26). These are also referred to as small peritoneal macrophages or bone marrow derived macrophages (14, 26). In the peritoneal cavity, PAI-1 and CCL1 were shown to recruit macrophages to the wound involving the receptor molecules CD11b and CCR8 respectively (116, 117). Depletion of macrophages using clodronate-loaded liposomes or genetic constructs results in decreased wound healing of sterile injuries of serosal surfaces of the liver and abdominal wall (36, 44). However, novel experimental strategies will be necessary to experimentally isolate the role of recruited macrophages compared to that of the resident GATA6+ macrophages in mesothelial wound healing. Furthermore, monocyte-derived macrophages have been shown to replenish the resident GATA6+ macrophages. This process is dependent on the transcription factor IRF4 (118). The degree of this replacement strongly depends on the degree of initial macrophage disappearance (50, 62). Taken together, this suggests a dual role of infiltrating monocytes in wound repair: they act as precursors of bone-marrow derived macrophages that are directly needed in wound repair, and they can serve to replace the GATA6+ resident macrophages.

Neutrophils and monocytes are not only recruited into the abdominal wall (45) but they are also recruited into the peritoneal cavity fluid. In fact, within a few hours after extensive mesothelial injury (surgery), large numbers of Gr1+ cells (neutrophils) and monocyte derived macrophages were recruited into the peritoneal cavity (119). This is consistent with observations in humans undergoing surgery where billions of neutrophils and monocytes can be isolated from the peritoneal fluid. This recruitment was proposed to be driven by chemokines such as MCP-1 and CXCL1 that were released into the peritoneal cavity by mesothelial cells in response to injury (119, 120). It is not known what molecules and mechanisms govern the migration of leukocytes from the interstitium, into the peritoneal fluid, where these cells adopt a planktonic form once again. Further, it would be interesting to investigate the migratory patterns of neutrophils and monocytes after they have reached their suspended state in the peritoneal cavity.

## Serosal (Mesothelial) Repair

So far, we have seen how a mesothelial injury leads to inflammation with the consecutive deposition of a fibrin matrix and infiltration of immune cells. Normal serosal repair is achieved when a) the underlying organ is repaired, b) the sub-mesothelial connective tissue layer is restored to its original composition and thickness and c) the integrity of the mesothelial membrane is reconstituted (31, 121, 122). The central role of the mesothelium in tissue repair and fibrosis has been revisited and comprehensively summarized (38).

The mesothelium is a slowly renewing tissue with less than 1% of cells undergoing mitosis at any time (123). After mesothelial injury, activated macrophages induce a pronounced proliferative expansion of the mesothelial compartment (124, 125). Genetic lineage tracing of mesothelial cells in several injury and disease models indicate that regenerating mesothelium originates from healthy mesothelium rather than submesothelial cells (126–128). Small focal mesothelial injuries that were induced using thermal probes on the liver capsule and abdominal wall, healed completely without any visible defect left after days (36, 44). Because repair of mesothelial defects is largely independent of the defect size, investigators have proposed that mesothelial cells not only crawl into the wound from the borders, but also detach from opposing surfaces and distant sites and migrate in a free-floating state through the coelomic cavity until they settle on the wound (125, 129, 130). This is further supported by the fact that adoptively transferred mesothelial cells improve mesothelial repair in the recipient (130). The response of mesothelial cells to injury can be summarized as proliferation, loss of epithelial cohesion and migration. These phenotypic changes are reminiscent of other serosal surfaces that undergo epithelial-to-mesenchymal transition (EMT). In analogy, this reaction has been termed mesothelial-to-mesenchymal transition (MMT) (131, 132). On a molecular level, MMT involves the downregulation of epithelial junctional proteins such as E-cadherin and the upregulation of mesenchymal marker α-smooth muscle actin (α-SMA) and production of ECM (133, 134). These changes correlate with an upregulation of transcription factors canonically associated with EMT such as SNAI1, SNAI2, ZEB1, ZEB2, Twist1 (38) and can be induced by exposing mesothelial cells to TGF-β1, hepatocyte growth factor, platelet derived growth factor and IL-1β (38). We will next discuss how mesothelial repair can become defective.

## Post-Surgical Adhesions

Surgeries within body cavities such as the abdominal cavity are often lifesaving procedures. Following surgical trauma, mesothelial repair can lead to a restitution of serosal surfaces at integrum. However, in some patients, the healing process is disrupted, leading to a fibrotic complication called post-surgical adhesions. Adhesions are fibrous bridges of various thickness and length containing blood vessels and nerve tissue (135, 136). Adhesions can also be caused by infection but today, surgeries comprise by far the most common cause of mesothelial injury leading to adhesions (96, 135). In the peritoneal cavity, adhesions result in considerable morbidity as they impair the free movement of organs. These problems include potentially life-threatening intestinal occlusions, secondary infertility in women, and chronic post-operative abdominal pain (96, 137, 138). Peritoneal adhesions were described for the first time in 1836 in a post-mortem examination of a patient that had died from peritoneal tuberculosis (139). It was then suggested in 1849 that these abnormal structures originate from lymphatic vessels that turn into fibrinous adhesions (135, 139, 140). Despite tremendous scientific advances since 1849 including the execution of many clinical and experimental studies on adhesions, understanding of their pathogenesis has not evolved enough to develop effective therapies. To date, few research and development resources are dedicated towards resolving this significant health problem. The process of adhesion formation largely depends on the same mechanisms as “normal” mesothelial repair: Healing is initiated by damage recognition, inflammation, and coagulation. These steps lead to the recruitment of leukocytes and the deposition of fibrin. Then, the stable fibrin matrix (fibrin clot) is infiltrated by myofibroblasts that start to deposit ECM proteins such as collagen.

**Figure 4:**
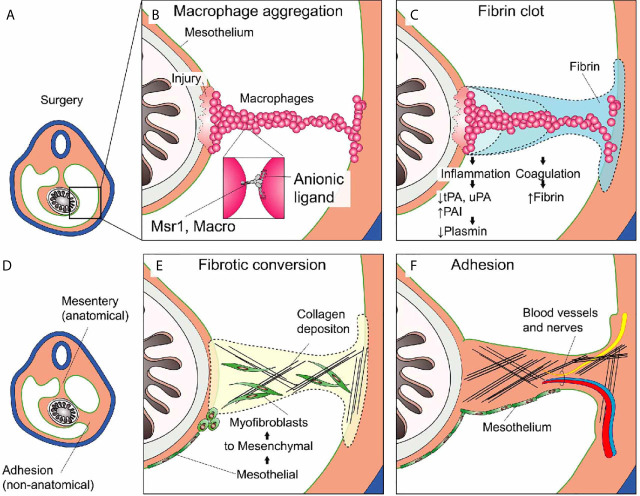
Post-surgical adhesion formation. **(A)** Overview of the peritoneal cavity before surgery. **(B)** Non-focal mesothelial injury such as major abdominal surgery leads to the uncontrolled aggregation of peritoneal macrophages serving as the nidus for the **(C)** subsequent Fibrin clot deposition. Inflammation and Coagulation inter-dependently promote the deposition of fibrin (see text). **(D)** Overview during or after adhesion formation. The abdominal organs (e.g., intestine) are now attached to the abdominal wall at anatomic (mesentery) and non-anatomic (adhesion) locations. **(E)** Mesothelial to mesenchymal transition gives rise to myofibroblasts that migrate into the wound and into the fibrin clot where they start to deposit extracellular matrix (ECM) such as collagen. **(F)** Adhesion formation is completed when the scar tissue is covered with mesothelium. The lesion may become fully perfused and pain-sensitive by ingrowth of blood vessels and nerves.

The problem with adhesions is that wound healing occurs at sites it should not (96). The classical paradigm of adhesion formation states that if serosal surfaces cannot re-establish homeostatic fibrinolysis soon after injury, excessive amounts of fibrin are deposited. We have discussed that macrophage aggregation accompanies fibrin deposition after sterile injury at serosal surfaces (3). Macrophage-fibrin deposits serve as the basic scaffold for tissue repair. Clots that span the space between opposing serosal surfaces are dangerous because they can be converted into scars that permanently link these surfaces called adhesions. We will now discuss the events taking place in more detail and highlight the respective therapeutic considerations for each (**Figure 4**).

### Macrophage Aggregation and Fibrin Clot Formation

The idea that mesothelial loss of baseline fibrinolytic activity after surgical trauma may cause adhesions has been the subject of various animal models (96). Interestingly, hypofibrinolytic fibrin deposition led to adhesion formation in many different injury models and different species (96) and became generally accepted as the “classical concept of adhesion formation” (96). We have seen that an exuberant inflammatory response induced by peritoneal injury or infection promotes an increased procoagulatory and antifibrinolytic reaction. On a molecular level, inflammatory mediators increase the expression of tissue factor (TF) and PAI-1 and decreases the expression of tPA in mesothelial cells resulting in increased fibrin deposition (96). This mechanism was confirmed in peritoneal biopsies of inflamed peritoneum of humans that underwent surgery. The reduction of fibrinolytic activity during inflammation was mediated by PAI-1 (96). Importantly, a prospective study in humans showed that PAI-1 concentrations in peritoneal fluid were correlated with the occurrence of adhesions after 8 days (96). Strategies to prevent postoperative adhesion target the dysregulated fibrin clot deposition by either inhibiting coagulation, increasing fibrinolytic activity, or reducing inflammation.

Administration of fibrinolytic (tPA) or anticoagulant agents (Heparin) significantly reduced adhesions in different animal models (38, 96, 141–144). However, the only study in humans that enrolled 102 patients, was unable to confirm this effect. In this study 5000 I.U. of heparin were diluted in saline and used to wash the peritoneal cavity. The patients in this study then underwent a second operation (laparoscopy) 12 days after the first, to obtain adhesion scores (145). The heparin dose that was effectively administered in these patients was not reported but it must have been extremely low as most heparin containing lavage solution was removed after a few minutes. Based on titration studies in an animal models, the threshold dose for significant anti-adhesion effect was (without occurrence of bleeding after two days) 7.5x10 U/kg/day, which is equivalent to 5250 I.U/day for a person weighting 70kg (142). Therefore, low heparin dose or low heparin concentration may be the reason no effect on adhesion formation was observed in the Jansen study. Further studies would be necessary. However, whether the current evidence justifies a human trial testing high dose heparin in major abdominal surgery remains to be discussed.

Others have tried to use anti-inflammatory agents to restore mesothelial fibrinolytic activity. Cyclo-oxygenase inhibitors and steroids were tested in animal models (146–148) with no resounding success. Experimental animal models demonstrate potent prevention of postoperative adhesion following intraperitoneal application of HMG-CoA reductase inhibitors (statins) (149). HMG-CoA reductase inhibitors stimulate fibrinolytic activity in human peritoneal mesothelial cell cultures (150) and exert anti-inflammatory functions (151). In experimental animal models amelioration of adhesion after administration of intraperitoneal acylated ghrelin, a 28-amino acid gastric peptide with anti-fibrotic and anti-inflammatory properties, was demonstrated. The adhesion prevention by ghrelin application was modulated *via* blockage of the TGF-β signaling pathway (152).

**Figure 5:**
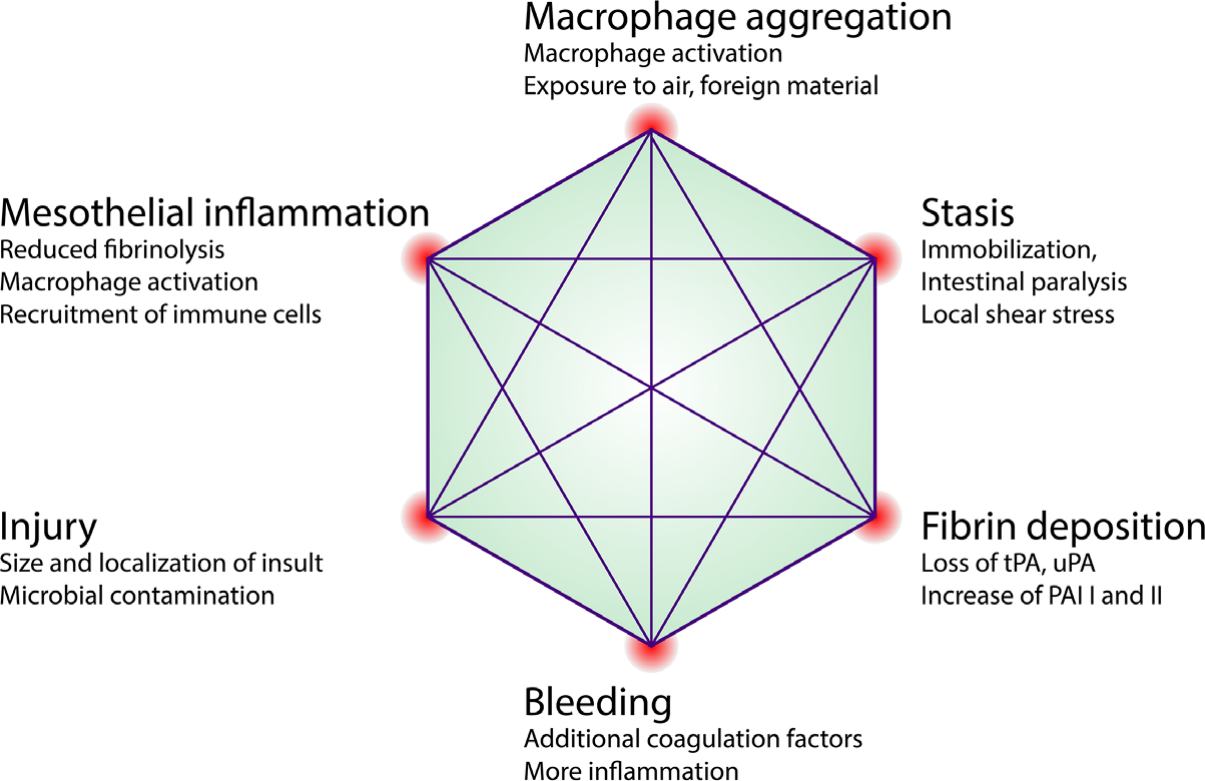
Factors influencing adhesion formation. Proposed concept of early local determinants that influence the binary outcome of adhesion formation and may be exploited therapeutically. tPA, tissue plasminogen activator; uPA, urokinase-type plasminogen activator; PAI, Plasminogen activator inhibitor.

Extending on this classical paradigm we have recently proposed yet another factor in adhesion formation: that of peritoneal macrophage aggregation. We showed that in macrophage aggregation to focal peritoneal injuries was tightly regulated with just enough cells aggregated to seal the defect. However, in response to large peritoneal injuries, such as abdominal surgeries, the aggregation of these macrophages was dysregulated, resulting in the formation of large super aggregates that started to join mesothelial surfaces. We found that this process was dependent on scavenger receptors MARCO and MSR1. Depleting peritoneal cavity macrophages or inhibiting their aggregation significantly reduced the amount and severity of adhesions in a mouse model. We therefore propose an adaptation of the classical paradigm to include peritoneal macrophage aggregation as an additional event (**Figure 4**). Before we discuss the later events in adhesion formation such as fibrotic conversion and remodeling, we would like to note that the early process of adhesion formation such as mesothelial inflammation (chapter 3), macrophage aggregation (chapter 3) and coagulation (chapter 4) are tightly linked. We have discussed how inflammation directly affects coagulation. Inversely, coagulation provides a positive feedback to the mesothelium further increasing inflammation. For example, activation of proteinase-activated receptor-2 (PAR2) on mesothelial cells results in increased MIP-2 production and consecutive neutrophil infiltration (153). Furthermore, peritoneal cavity macrophages were shown to produce coagulation factors (21, 23, 154) and Factor V produced by peritoneal macrophages was shown to be essential for the clotting of peritoneal fluid in response to bacteria (21). Inversely, they showed that macrophage aggregation (disappearance) was partially dependent on coagulation factors. In other models such as laser-induced sterile mesothelial injury, the aggregation of macrophages was largely independent of fibrin crosslinking and macrophages showed the ability to aggregate ex vivo without addition of fibrin (44). Taken together, mesothelial inflammation, macrophage aggregation, and coagulation, can act cooperatively but do not necessarily depend on each other. The relative contribution of each of these three processes likely depends on the type (sterile, microbial, combined) and strength of the insult as well as on local shear (3), which in turn is largely dependent on patient movement and post-surgical intestinal paralysis (**Figure 5**).

### Fibrotic Conversion

Alpha smooth muscle actin (α-SMA) positive myofibroblasts are considered the main collagen-producing cell in wound healing and many fibrotic diseases (155–157). The question of the origin of α-SMA positive myofibroblasts in adhesions has been a matter of debate. Myofibroblasts in adhesions were believed to be either derived from the mesothelium or alternatively derived from sub-mesothelial cells (126). Recently, Fischer at al. used a genetic fate mapping to permanently and selectively label cells expressing protein c receptor gene (*Procr^CreERT^ x Rosa26^tdTomato^*). Tamoxifen administration in these reporter mice resulted in the selective and permanent labelling of approximately 50% of all mesothelial cells but not submesothelial cells (127). Using this approach, they were able to show that the majority of platelet derived growth factor receptor α positive (PDGFRα+) myofibroblasts in adhesions were of mesothelial origin (127). This is in line with older studies that relied on non-genetic lineage tracing methods such as cell tracker dyes or lineage markers to infer on source of myofibroblasts in adhesions (122, 158, 159). Taken together these data suggest that mesothelial to mesenchymal transition (MMT) is the major source of myofibroblasts in adhesion pathogenesis. On a molecular level, MMT in adhesion formation relies on the same pathways as mesothelial repair (159). In fact, administration of TGF-β blocking peptide P144 resulted in a significant reduction of adhesions in an experimental mouse model (159). This was associated with a reduced expression of MMT markers such as Snail, α-SMA and Collagen I in P144 treated mice (159). In addition, the exposure of mesothelial cells to cyclic mechanical forces was shown to increase MMT in experimental murine and human models. Biomechanical induction of MMT cooperates with biochemical signals such as TGF-β and seemed to be regulated by caveolin-1, a plasma membrane mechanotransducer (160). Interestingly, MMT-cells in adhesions also express many markers that are found in the mesothelium during embryonic development but not within the adult mesothelium. These markers, including Mesothelin (MSLN), Uroplakin-1B and Wilms-tumor 1 (WT1), were upregulated in adhesions indicating that adult mesothelial cells can repurpose aspects of fetal development (158). Depletion of mesothelial cells results in a complete reduction of adhesions (127). However, totally depleting the peritoneal cavity of potential myofibroblasts may compromise wound healing too much for use in the clinic. Strategies that inhibit MMT in adhesions but leave mesothelial repair intact need to be developed.

### Remodeling

After fibrotic conversion, adhesions are considered irreversible and redundant scar bands. Furthermore, animal, and human studies demonstrated the ingrowth of nerves and vessels into adhesions. In a murine model, nerve fibers in abdominal adhesions were detected already two weeks after surgery and 4 weeks post-surgery the nerve fibers were traversing the whole adhesion from coecum to abdominal wall (161). Human peritoneal adhesion specimens collected during surgery from 25 patients contained invariably sensory nerves (162). The sensory innervation could partially explain the chronic pain a lot of patients with adhesions experience. Animal studies revealed blood vessels in adhesions already 6 hours after injury (163). This process of remodeling from connective tissue to fully innervated and vasculated tissue might be modulated.

The nerve fibers in adhesions were often associated with blood vessels indicating angiogenesis could play a key part in regulating ingrowth of nerves into adhesions (162, 164). Local production of VEGF by mesothelial cells appears to play a central role in the process leading to peritoneal angiogenesis (165). In different murine models, postsurgical adhesion formation was reduced by inhibition of VEGF suggesting adhesion formation is angiogenesis-dependent (166, 167). In a human study including adhesions samples from patients years after first surgery, adhesions expressing VEGF A and its receptor showed significantly higher numbers of immature vessels suggesting ongoing angiogenesis in mature adhesions (163). In addition to angiogenesis, modulation of the ECM by matrix metallo-proteinases (MMPs) takes place. MMPs are proteolytic enzymes involved in degradation of ECM, their activity is opposed by tissue-derived inhibitors of MMPs (TIMPs) (168). The expression of both VEGF and MMPs is upregulated during MMT (131). In a human study, peritoneal samples were collected during initial laparoscopy and during a second-look laparoscopy 48 hours later. Patients with pelvic adhesions exhibited significantly lower amounts of MMP-9 concentrations and significantly higher MMP-9/TIMP-1 ratios when compared with controls (169). In peritoneal fluid of patients with excessive adhesions, higher TIMP-1 levels could be demonstrated compared with those of patients without adhesions (170). Mice treated with instillation of adenovirus vector encoding mutant MMP-9 gene at the time of peritoneal injury showed a reduced of severity of *de novo* adhesions (168). It remains unclear whether adhesion keep their capacity for remodeling and thus have the potential to spontaneously resolve, or whether adhesions are an irreversibly fixed pathology once they have developed.

## Concluding Remarks

We have discussed how mesothelial repair works for small injuries and how it can go wrong and result in peritoneal adhesions. It is important to note that mesothelial repair plays an important role in other fibrotic disorders in proximity to serosal surfaces. These disorders include the fibrotic thickening of the peritoneum (peritoneal fibrosis or encapsulating peritoneal sclerosis) and the pleura (pleural fibrosis). A number of studies have also demonstrated that mesothelial cells play also an important role in fibrotic diseases of the liver (128) and lung (38, 171–173). This is not surprising, since both, the liver and the lungs are covered with visceral mesothelium. In these disorders the mesothelial cell plays an important role and similar mechanisms are at play. Overall, there are many questions that need to be addressed to improve our understanding of wound healing at serosal surfaces which in turn might have great impact on the way we think of any of the above-mentioned pathologies.

## Author Contributions

JZ and SNZ wrote the article. DS gave substantial input to the concepts underlying this review and revised and approved the article.

## Funding

JZ was supported by a Swiss National Science Foundation (SNSF) research fellowship (P1BEP3_181641).

## Conflict of Interest

JZ is holding patent rights (US63/125,020) for the use of scavenger receptor inhibitors to treat post-surgical peritoneal adhesions.

The remaining authors declare that the research was conducted in the absence of any commercial or financial relationships that could be construed as a potential conflict of interest.
